# A Second Genome Sequence of an Enterovirus C99 Detected in a Healthy Chimpanzee

**DOI:** 10.1128/MRA.00893-20

**Published:** 2020-10-15

**Authors:** Illich Manfred Mombo, Larson Boundenga, Nicolas Berthet, Rebeca Atencia, Debby Cox, Gael Darren Maganga, Christiane Bouchier, Eric Maurice Leroy, Virginie Rougeron

**Affiliations:** a Centre International de Recherches Médicales de Franceville, Department of Virology, CIRMF, Franceville, Gabon; b Unité Environnement et Risque Infectieux, Cellule d’Intervention Biologique d’Urgence, Paris, France; c Tchimpounga Chimpanzee Rehabilitation Centre, The Jane Goodall Institute, Pointe-Noire, Republic of the Congo; d Institut National Supérieur d’Agronomie et de Biotechnologies (INSAB), Université des Sciences et Technique de Masuku, Franceville, Gabon; e Institut Pasteur, Genomic Platform, Paris, France; f Laboratoire MIVEGEC UMR 5290, CREES, CNRS, Institut de Recherche pour le Développement, Université de Montpellier, Montpellier, France; KU Leuven

## Abstract

We report the nearly complete genome sequence of an enterovirus 99 strain (Cpz-IJC08) detected in a healthy chimpanzee from the Tchimpounga Sanctuary in the Republic of Congo. According to the phylogeny, Cpz-IJC08 clustered with Cpz-IJC04, a previously identified chimpanzee enterovirus from the same sanctuary, isolated from an animal with signs of acute flaccid paralysis.

## Abstract (precis)

We report the nearly complete genome sequence of an enterovirus 99 strain (Cpz-IJC08) detected in a healthy chimpanzee from the Tchimpounga Sanctuary, Republic of Congo. According to the phylogeny, Cpz-IJC08 clustered with Cpz-IJC04, a previously identified chimpanzee enterovirus from the same sanctuary, isolated in animals with signs of acute flaccid paralysis.

## ANNOUNCEMENT

Enteroviruses (EVs) are positive-sense single-strand RNA viruses of the genus *Enterovirus* (*Picornaviridae*). Their genomes are approximately 7.5 kb and encode a single polyprotein flanked by 5′ and 3′ untranslated regions (UTR), including the capsid protein (P1) and the nonstructural coding regions (P2 and P3). EVs, transmitted via the fecal-oral route, are responsible for mild to severe illnesses such as the common cold; gastroenteritis; hand, foot, and mouth disease; and acute flaccid paralysis (AFP) (1). The genus *Enterovirus* is composed of 15 species, and some serotypes (e.g., EV-C99) belonging to the species EV-A, EV-B, EV-C, and EV-D infect humans and nonhuman primates (NHPs) (2). Even though EV-C99 has been detected in various NHP species (2, 3), there is currently only one genome available.

In a previous study, we reported the characterization of an EV-C99 in a chimpanzee from the Tchimpounga Sanctuary in the Republic of Congo, presenting AFP clinical signs during an AFP outbreak in humans in 2010 (3). Here, we report another genome of serotype EV-C99 from a sample taken from another chimpanzee individual (IJC08) from the same group collected in the same period; this individual did not present any AFP signs.

The stool sample of the chimpanzee was suspended in phosphate-buffered saline (PBS), and total RNA was extracted from 400 µl of the supernatant using an EZ1 Advanced XL instrument (Qiagen) according to the manufacturer’s procedure. The extracted RNA was treated with Turbo DNase (Life Technologies). A reverse transcription reaction was performed using the SuperScript III first-strand cDNA synthesis system (Life Technologies) using random hexamers and the Phi29 enzyme (QuantiTect whole-transcriptome kit; Qiagen) (4). The cDNA library was constructed using the NEBNext Ultra II kit (NEB). Briefly, amplified DNA was fragmented into 300 to 1,000 bp using a Covaris ultrasonicator, and sequencing was performed on a HiSeq 2000 device using the 101-base single-end TruSeq 50-cycle sequencing-by-synthesis (SBS) V3 kit (Illumina).

A total of 17,371,302 reads were obtained from Cpz-IJC08. Reads with an average Phred score value below 20 and shorter than 200 bp were removed. Viral reads were selected using BLASTN, and only those matching the EV-C99 genome were considered (5). Selected reads were assembled with ABySS V3.5 (6), and contigs were then assembled into scaffolds with the CAP3 V1.0 program (7) to obtain the complete genome sequence. We ended with 55,022 reads *de novo* assembled and a viral genome of 6,648 nucleotides (nt), with a GC content of 44%. Despite repeated attempts, 5′ and 3′ UTR were not obtained. The Cpz-IJC08 genome encodes a single open reading frame (ORF) encoding 2,210 amino acids.

**FIG 1:**
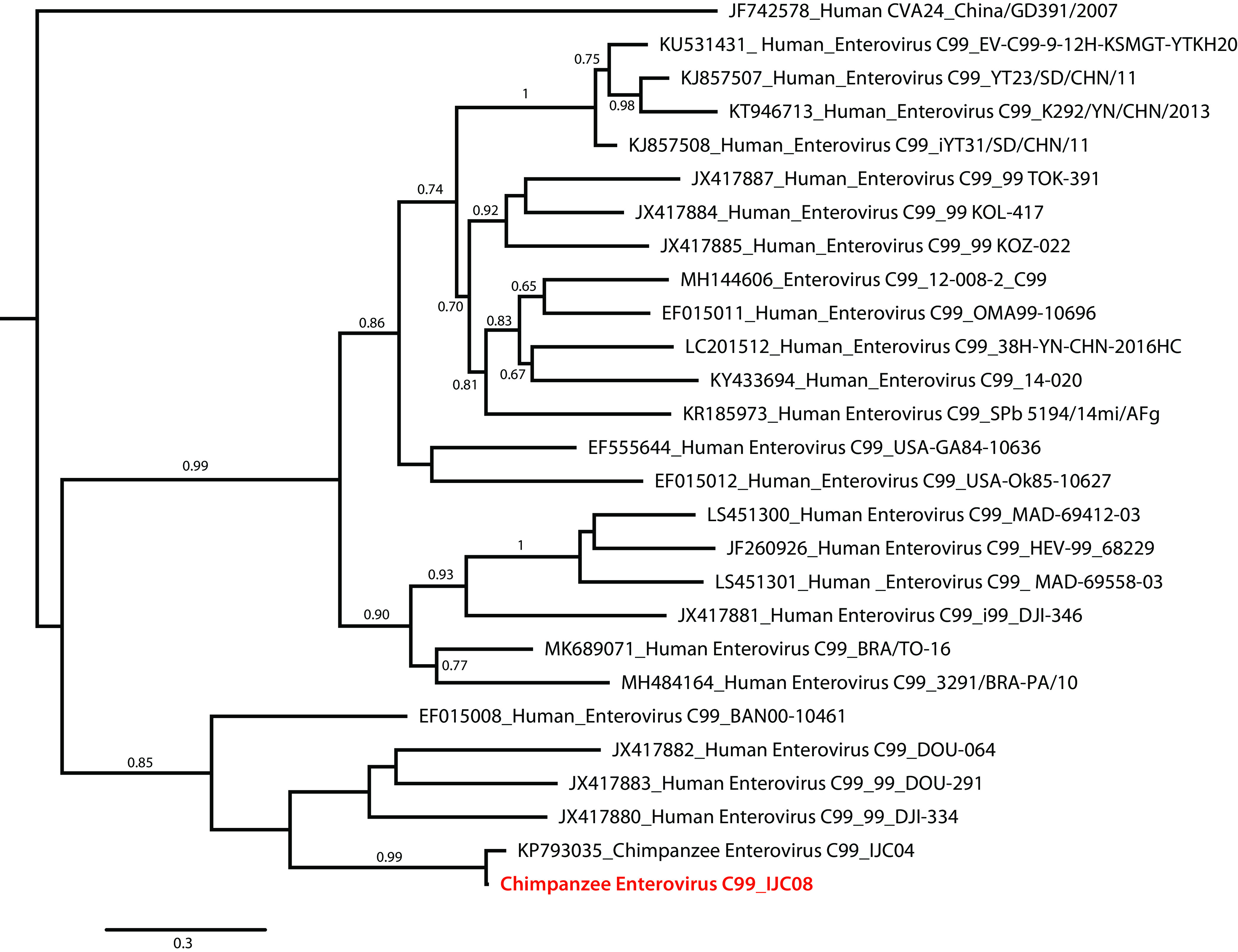
Maximum likelihood phylogenetic analysis based on the complete VP1 nucleotide sequence. The multiple sequence alignment was performed using the ClustalW algorithm implemented in the MEGA7 V7.0.36 software package (8). Phylogenetic trees were constructed using the maximum likelihood method (freely available at the www.phylogeny.fr bioinformatics platform [9]) using the general time-reversible (GTR) model of branch support (10) and 1,000 bootstrap replicates. Only bootstrap values above 0.6 are indicated on the branches. Accession numbers and strain or isolate names are included in the taxon labels. The complete genome of this study is indicated in red. All tools were run with default parameters unless otherwise specified.

The phylogenetic analysis based on the complete VP1 gene showed that Cpz-IJC08 clustered most closely with strain Cpz-IJC04 (97.7% nucleotide similarity; GenBank accession number KP793035) and more distantly (73.4% to 81.5%) with the other strains (Fig. 1).

This study reports the second genome of an EV-C99 strain obtained from a healthy chimpanzee, indicating the natural circulation and the asymptomatic carrying of such strains in this NHP species. Such genomic information contributes to improving our understanding of phylogenetic relationships between EV strains circulating in primates.

### Data availability.

The Cpz-IJC08 genome has been submitted to GenBank and the SRA under accession numbers MT569434 and PRJNA648789, respectively.
